# Facing further challenges in cancer data quality and harmonisation

**DOI:** 10.3389/fonc.2024.1438805

**Published:** 2024-07-25

**Authors:** Francesco Giusti, Carmen Martos, Raquel N. Carvalho, Vesna Zadnik, Otto Visser, Manola Bettio, Liesbet Van Eycken

**Affiliations:** ^1^ European Commission, Directorate General Joint Research Centre, Ispra, VA, Italy; ^2^ Belgian Cancer Registry, Brussels, Belgium; ^3^ Foundation for the Promotion of Health and Biomedical Research in the Valencian Region (FISABIO), Valencia, Spain; ^4^ Slovenian Cancer Registry, Institute of Oncology Ljubljana, Ljubljana, Slovenia; ^5^ Department of Registration, Netherlands Comprehensive Cancer Organisation (IKNL), Utrecht, Netherlands

**Keywords:** cancer registry, data quality, data harmonisation, challenges, Europe

## Abstract

This article highlights the recent and ongoing activities of European population-based cancer registries (PBCRs) in data quality and harmonisation in the framework of the collaboration between the European Network of Cancer Registries (ENCR) and the Directorate-General Joint Research Centre (JRC), the science and knowledge centre of the European Commission. The article concludes the Frontiers in Oncology’s Research Topic “Joining Efforts to Improve Data Quality and Harmonization Among European Population-Based Cancer Registries”, which has been an opportunity for several European researchers to share their experience on cancer data quality and harmonisation. Such experience will be helpful for PBCRs in view of future challenges and opportunities in cancer epidemiology, with a few examples discussed in the present article.

## 1 Introduction

During recent decades, the role of population-based cancer registries (PBCRs) has advanced beyond their traditional focus on providing cancer incidence and survival data, enlarging it to data providers for health-service management (1–4). In this respect, PBCRs face further challenges of data quality and harmonisation issues.

Since 1990, the European Network of Cancer Registries (ENCR) has been operational with the aim to connect PBCRs in Europe.

The ENCR plays a crucial role in supporting PBCRs to improve the quality (including comparability) and availability of cancer incidence data and paves the way for the use of data collected by PBCRs in cancer control, health-care planning and research. Cancer data comparability between countries and regions is particularly important for the European policy makers, who rely on the European Cancer Information System for accurate and up-to-date cancer burden statistics computed with data from the almost 200 PBCRs currently active in Europe. ENCR activities have a global impact, also due to its collaborations with the International Association of Cancer Registries (IACR) and the International Agency for Research on Cancer (IARC) and the fact that ENCR recommendations and guidelines regularly serve as models endorsed within the IACR. An example of collaboration between ENCR and IACR was the joint ENCR-IACR 2023 Scientific Conference, which took place in Granada, Spain, in November 2023 and was attended by more than 350 participants (5).

The *Frontiers in Oncology* Research Topic “*Joining Efforts to Improve Data Quality and Harmonization Among European Population-Based Cancer Registries*” has been an opportunity for European researchers to share their experience on cancer data quality and harmonisation (6).

In this light, this article refers to all the contributions to the Research Topic and summarises the present situation in European PBCRs related to data quality and harmonisation, as well as the currently implemented activities carried out by ENCR and JRC to improve them. Of particular note, the activity of several ENCR working groups and the update of ENCR recommendations will be described. Moreover, the European Cancer Information System (ECIS) (7) as the ultimate outcome of data quality and harmonisation efforts will be presented.

## 2 Current advances of cancer registration in Europe

Since 2012, the ENCR Secretariat has been hosted at the Joint Research Centre (JRC), the science and knowledge centre of the European Commission. In this scenario, several initiatives were carried out in the last decade (8) aimed at improving cancer data quality and harmonisation of European PBCRs: the JRC and ENCR coordinated thematic expert working groups to draft guidelines and recommendations on data collection, coding, and reporting, organised trainings, including on the revised recommendations, and developed common rules and related validation software to check data compliance to agreed European standards (9).

European PBCRs are very heterogeneous in terms of geographical coverage, either national or regional, and can cover very different population sizes, translating in datasets ranging from around 125,000 to over 50 million cancer records. Additionally, they differ regarding registration practices, for example in relation to data sources, definitions and procedures. Therefore, common rules and definitions are necessary in order to harmonise data from different PBCRs and ensure their comparability at European level.

To this purpose, the following recommendations, reports and documents were published during the period 2022–2024 on the ENCR website (10).

### 2.1 ENCR recommendations

#### 2.1.1 Data quality checks for European cancer registries

Recognising the pivotal importance of comparability, completeness, validity, and timeliness in ensuring the reliability and utility of PBCR data, in 2013 the ENCR and JRC launched the Data Quality Checks Working Group to address the fragmented landscape of data validation methods across European PBCRs.

To achieve this objective, a series of workshops were convened in 2013 and 2014. These meetings served as forums for stakeholders from diverse backgrounds, including PBCR experts, epidemiologists, and data analysts, to collaboratively deliberate on the establishment of a harmonised framework for data quality assessment.

Following the work of the Cancer Data Quality Checks Working Group (11) the first agreed quality control checks among European PBCR’s were proposed, aimed at validating the internal consistency of cancer incidence variables. The report, and later update (12) formed the basis for the JRC-ENCR Quality Check Software (QCS), described in one contribution of the current Research Topic (13).

#### 2.1.2 Standard dataset for the European network of cancer registries (2023)

This recommendation updates a previous document released in 2005 (14), to provide the minimum dataset to be collected by European PBCRs. Given the great expansion of PBCRs role in cancer control, quality assessment of cancer care, clinical and epidemiological research in the latest years, additional standardised data items were deemed necessary for registration. Thanks to the rapid growth of electronic records in the health care sector, many items may now be collected by linkage to existing data sources, as part of routine operations or on an *ad hoc* basis. However, the abundance of available data may be at the expense of standardisation and comparability. While the level of automation may increase access to growing amounts of data, the legal basis for access to and linkage with health data, varying greatly across Europe, may jeopardize the capacity to check the quality of such data.

The 2023 revision of the standard dataset recommendation (15, 16) was drafted to preserve the possibilities for comparisons on cancer incidence between European and non-European PBCRs, to share data definitions for in-depth and wide-scale collaborative efforts and identify variables that may support an expanded role of PBCRs in cancer control.

#### 2.1.3 Basis of diagnosis (2022)

The 2022 recommendations updated the previous ones from 1999 (17–19).

Basis of diagnosis is a key variable, including information both on the way in which the tumour is diagnosed and the level of likeness of the diagnosis itself. It is also influenced by the ability of individual PBCRs to intercept the different (pathological, cytological, molecular…) reports.

Guidance in the latest recommendations is particularly relevant in the absence of pathological confirmation of the tumour. The proportion of clinical diagnoses (basis of diagnosis values 1, 2 and 4) is a data quality indicator. While a high proportion of clinical diagnoses in a PBCR may reflect the situation with regard to clinical and pathological investigations in the area covered by the PBCR, it may also indicate overdiagnosis and overestimation of cancer incidence, possibly taking into account tumours that would never have caused symptoms or death. On the other hand, PBCRs with a very low proportion of clinical diagnoses might underestimate incidence rates, potentially missing cancer cases that should be counted.

Among the modifications introduced, the new value 8 (Cytogenetic and/or molecular testing) for coding the basis of diagnosis is particularly relevant in view of the fast evolution of diagnostic techniques, such as karyotyping, FISH (fluorescent *in situ* hybridization), PCR (polymerase chain reaction) and DNA sequencing.

#### 2.1.4 Cancer cases in migrant population (2022)

In the wake of the increase in the number of migrants (including refugees) in European countries, and with a particular consideration of the millions of refugees from Ukraine to Europe, in 2022, a new ENCR recommendation was released to clarify and harmonise whether to register migrant individuals without a legal residency at the date of incidence (20).

#### 2.1.5 Recording and reporting of urothelial tumours of the urinary tract (2022)

Following the previous publication in 1995 of “*Recommendations for coding bladder cancers*” (21) and IARC’s 2003 book on “*Standards and guidelines for cancer registration in Europe*” (22), knowledge about the biology and pathology of urinary tract tumours and their classification has increased considerably (23). Great variability has been observed among European PBCRs in the recording (i.e. registration) and the reporting (i.e. in presenting cancer burden statistics) of these tumours (24).

The 2022 ENCR recommendation aimed at improving comparability of data on urothelial tumours of the urinary tract in Europe by defining criteria mainly for registration, taking into account multiple aspects of these tumours such as primary topography, histological type, grade, extent of invasion, multi-centricity, progressions and time interval between tumors (25, 26). An example of the rules that should lead to greater data harmonisation and comparability is the suggestion not to record the “Urothelial proliferation of uncertain malignant potential”, which in any case are not reportable.

#### 2.1.6 Coding incidence date (2023)

The previous recommendation on the coding of incidence date was released in 1995 and revised in 1997 (27). The detection of inconsistencies in its application among European PBCR’s led to the creation of a working group which re-prioritized events considered for the registration of incident date considering modern methods of diagnosis such as flow cytometry, molecular testing, screening tests and more recent radiological and imaging techniques (28). An increased standardisation of incidence date, in addition to allowing more accurate cancer incidence statistics, also improves the consistency of survival estimates.

#### 2.1.7 ENCR endorsement of the Toronto childhood cancer stage guidelines (2016)

In 2016 the ENCR Steering Committee endorsed and encouraged the active use of the Toronto Childhood Cancer Stage Guidelines by European PBCRs, in order to promote the consistency of stage data for childhood malignancies (29–31). Moreover, the Toronto childhood cancer stage has been included in the latest 2022 ECIS data call protocol to European PBCRs.

One article of the current Research Topic shared the experience of the International Benchmarking of Childhood Cancer Survival by Stage (BENCHISTA) project in encouraging the implementation of the Toronto Childhood Cancer Stage Guidelines (32). The extensive application of the Toronto staging allows for instance to study whether the differences in survival of patients with childhood cancers between countries are due to a different diagnostic timing or to differences in access to care and treatment protocols, which is the main objective of the BENCHISTA project.

### 2.2 The European cancer information system

The JRC has been developing since 2012 ECIS as a comprehensive infrastructure, consisting of several components to manage a central data repository and to coordinate in an efficient and sustainable way the activities of data validation, analysis, and dissemination. A key component of the ECIS is a web-based tool launched in February 2018 (33) to report and disseminate cancer burden indicators such as incidence, mortality, survival and prevalence. Indicators in ECIS are derived from European PBCRs data. The ECIS web application (34) allows the visualisation of such indicators across European areas and time dimension.

The first data call to feed ECIS was launched in 2015. The database feeding ECIS is dynamic and is updated as new data becomes available.

The ECIS web-application is modular and currently, its data explorer section consists of the following modules:

- *Incidence and mortality estimates*– latest release year is 2022 as the outcome of a collaborative project between JRC and the IARC, in collaboration with the ENCR;
- *Long-term incidence and mortality estimates up to 2040*, evaluating the impact of different demographic scenarios by 2040 on the cancer burden;
- *Survival estimates*, reporting on the results of the latest published EUROCARE-5 study (35);
- *Incidence and mortality historical data*, including indicators computed from PBCRs observed data;
- *Childhood cancer incidence historical data*, reported according to the International Classification of Childhood Cancer (ICCC), third edition.
- Prevalence estimates in 2020, reporting on the results of the EUROCARE-6 study (36).

#### 2.2.1 The 2015 Call for Data protocol

The 2015 Call for data protocol required the submission from European PBCRs of a cancer case file, a population file, a mortality file, life tables and a data submission questionnaire (37). Data were harmonized at central level, incidence and mortality indicators were computed by the JRC and disseminated through the ECIS web application, released in 2018.

The variables required by the protocol for the incidence file included demographic and tumour characteristics like sex, age, topography, morphology, considered as core variables for reporting incidence indicators and which were the focus of data quality evaluations. Additionally, the protocol included variables for survival analysis, as well as stage and treatment information.

#### 2.2.2 The 2015 European dataset

**Figure 1:**
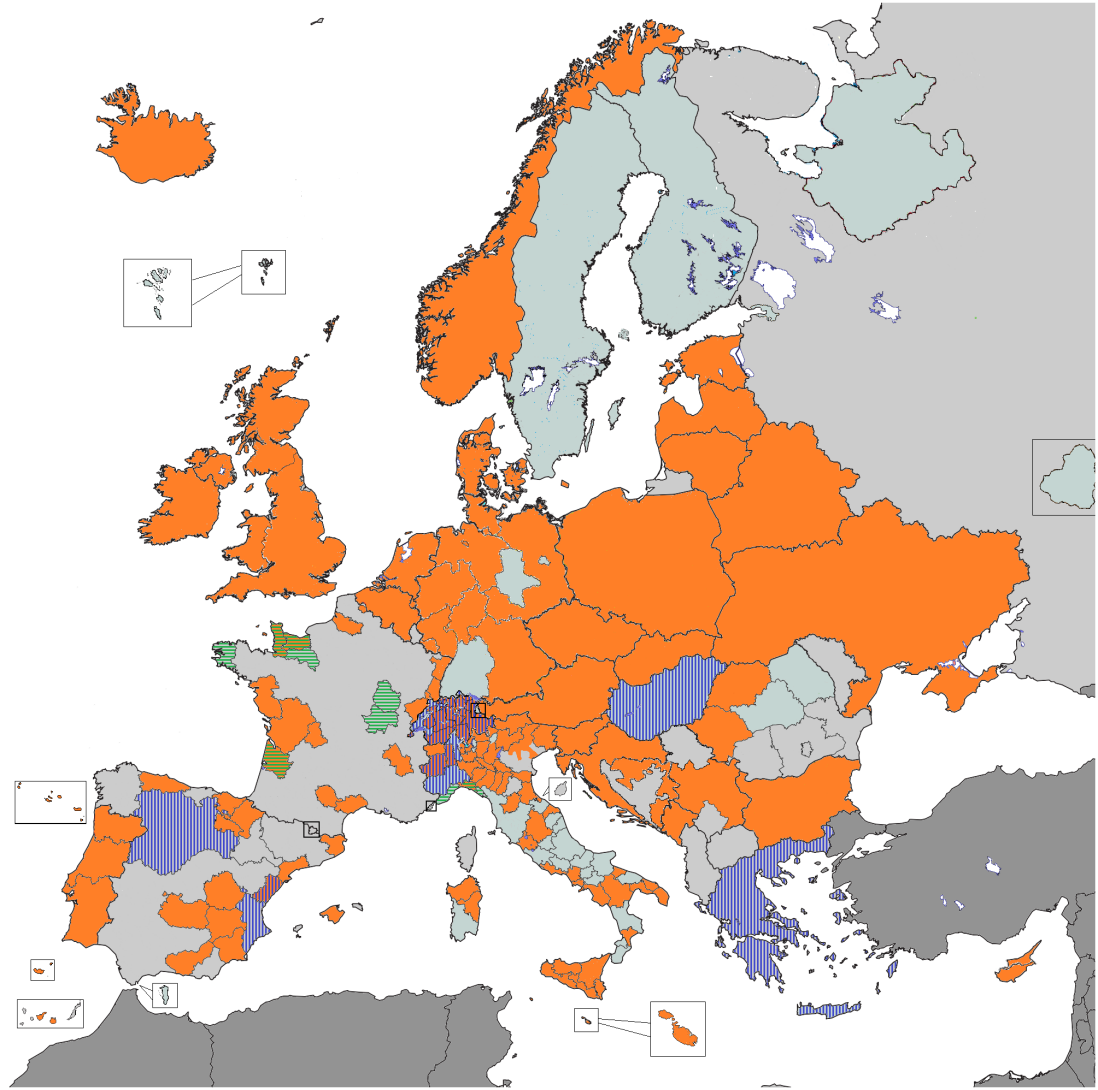
PBCRs contributing data 2015 ENCR-JRC Call for Data. Orange: all ages and all cancer sites PBCRs; Vertical stripes: childhood PBCRs; Horizontal stripes: site-specific PBCRs.

ENCR-affiliated PBCRs contributed data to the 2015 Call for Data (**Figure 1**). Over 34.5 million incident cases were collected from general PBCRs (all ages and all cancer sites) and specialised (childhood or site specific) PBCRs. Data harmonisation procedures, such as correction of errors detected by the JRC-ENCR Quality Check Software and the implementation of multiple primary tumours rules were performed centrally at JRC and by the submitting PBCRs. Following data harmonisation, around 30 million cases from 145 PBCRs (with incidence years between 1953 and 2014) were validated for the ECIS web application.

#### 2.2.3 The 2022 data call protocol

A second ECIS call was launched in 2022 to the ENCR PBCRs (38). While the core variables from the 2015 protocol were retained, the experience gained from the previous call led to a few changes in the 2022 protocol, namely:

- the case definition was changed: in situ/non-invasive tumours requested only for breast, urothelial tumours, ovary and skin melanoma, whereas, according to the ICD-O-3.2 the only benign tumours should be those of the central nervous system and gastrointestinal stromal tumours (GIST);
- A finer geographical detail was requested, specifying the geographical area of residence at diagnosis for incident cases according to the NUTS classification level 2 (NUTS2) (39);
- Toronto childhood cancer stage was introduced;
- Better specification of treatment (e.g. for different systemic therapies) and related timing (e.g. neo-adjuvant vs adjuvant) was added.

Novelties in the 2022 ECIS protocol implied the definition and implementation of additional validation rules, and related work for the update of IT tools.

### 2.3 Data quality aspects addressed in the current research topic

As shown in the present Research Topic, the quality of incidence data reported by European PBCRs improved between 1995 and 2014 (40). The analysis of 28,776,562 cases from 130 PBCRs in 30 European countries reported worse data quality for the oldest age groups and for cancer sites with poor survival. No differences were found between males and females, whereas high variability in data quality was detected across European PBCRs. The use of electronic health records, steadily increasing over the years, might be one of the contributing factors for a more accurate and timely registration of data.

A second contribution of the Research Topic focused on geographical variability and data quality in gastric and oesophageal cancer. A wide variability in oesophago-gastric cancers topographic subsites and histopathological types was observed, with a corresponding improvement in accuracy of registration in the study period (1995–2014) (41).

One article of the Research Topic focuses on the JRC-ENCR Quality Check Software (QCS) (13), as the IT tool developed by the JRC to check the internal consistency of PBCRs data.

Another valuable article of the present Research Topic thoroughly compared the functional characteristics of the JRC-ENCR QCS with the check tool developed by the IARC and the IACR (42). The paper concluded that it would be advisable to use both systems for data quality control, since they provide checks on different groups of variables (stage, follow-up) or on the same variables but with different modalities.

Finally, one important aspect of the improvement in data quality in European PBCRs is related to the enhanced possibility to analyse long-term cancer incidence trends. One example of such investigation is the article focusing on the incidence pattern of haematological neoplasms in Spanish children between 1983 and 2018, and its comparison with other southern European countries (43).

### 2.4 Current focus of JRC- ENCR activities

Harmonisation activities continue to be one major focus of the collaboration between the ENCR and the JRC. More specifically, the following topics are the subject of active ENCR Working Groups (9):

#### 2.4.1 Working group on treatment data harmonisation

As reported in the present Research Topic, a growing number of European PBCRs are collecting treatment data (44). This overview, which combined data from a literature review and conference proceedings, together with data from 125 European PBCRs, has led to the creation of a working group which provided the first recommendations for treatment data collection and coding, and the invitation to PBCRs to improve data harmonisation and comparability in Europe.

#### 2.4.2 Working group on cancer recurrences

The aim of the working group is to define a protocol for the standardised collection of cancer recurrence, progression and transformation data by PBCRs.

#### 2.4.3 Working group on central nervous system tumours

Aimed at updating the previous ENCR recommendation, dated 1998.

#### 2.4.4 Working group on haematological malignancies

Aimed at updating the previous ENCR recommendation, dated 2014.

#### 2.4.5 Working group on survival in ECIS

Aimed at defining the data standards and quality checks to be applied for publication of survival indicators in ECIS.

#### 2.4.6 Working group on multiple primaries registration

Aimed at updating the previous ENCR recommendation, dated 2004.

### 2.5 ECIS in the context of the European commission’s Europe’s Beating Cancer Plan

The European Commission’s Europe’s Beating Cancer Plan (EBCP) (45), released in February 2021, is structured around four key action areas (Prevention, Early detection, Diagnosis and treatment, Improvement of quality of life) and is supported by 10 flagship initiatives, underscoring the European Union’s commitment to support cancer prevention, treatment, and care across the continent. In this context, a Knowledge Centre on Cancer (46) and the European Cancer Inequalities Registry (47) were established in the framework of the EBCP.

Several activities and collaborations are ongoing to expand the information provided by the ECIS in line with commitments of the EBCP and demand for good quality data at population level will continue. The following is a list of developments in line with such commitments, including:

- Providing cancer incidence data at regional level, following the NUTS (Nomenclature of Territorial Units for Statistics) classification level. The availability of more granular data will facilitate ecological comparisons (for instance, with environmental and socio-economic data) and align with the overarching EBCP actions aiming to address inequalities between and within EU Member States. On this point, it will be important to monitor possible issues of reidentification of patients that might arise with more granular data;
- Displaying of cancer prevalence data, necessary for proper quantification to support EBCP objectives of reducing the burden of cancer, improving cancer outcomes, and enhancing the quality of life for all cancer survivors across Europe;
- Reporting on cancer stage data, which guide evidence-based decision-making tracking advancements towards cancer control goals and promoting quality improvement in cancer care;
- Exploring the expansion to cancer screening data monitoring, in line with the fourth EBCP flagship initiative, which aims to put forward a new EU-supported Cancer Screening Scheme to help Member States ensure that 90% of the EU population who qualify for breast, cervical and colorectal cancer screenings are offered screening by 2025. The CanScreen-ECIS project (48) paved the way towards this achievement.

## 3 Discussion

The improvement in quality and the harmonisation of PBCRs data will remain the focus of JRC and ENCR activities. As indicated by European PBCRs (49), a priority should be to develop a common mechanism for estimating the national cancer burden for countries with partial cancer registration, to enable direct and more accurate comparisons between countries. In addition, countries with absent or underdeveloped cancer registration should be assisted in establishing PBCRs. The quality indicators reported in the present Research Topic can be used as the baseline for monitoring PBCRs data quality indicators in Europe (40).

Reliable data from PBCRs are crucial for the effective implementation and evaluation of cancer control programmes. The standardisation of data and the harmonisation of procedures has led to an overall improvement in the description of neoplastic diseases and how incidence, survival, prevalence, mortality are all necessary (and somehow interlaced) indicators for understanding the epidemiology of tumors. The role of PBCRs has been expanding over the years; at the same time, thanks also to the essential action of ENCR and JRC, European PBCRs have made progress over the last decades with regard to data quality. This momentum should be sustained in order to further improve harmonisation and decrease resource disparities leading to quality disparities. Clear guidelines and policies offer the basis for this, with guiding principles for the equitable and effective operation of PBCRs providing a structured framework that enables registries to maximise their potential and contribute to cancer surveillance and research efforts, regardless of resource constraints.

Ongoing advances in technology can offer alternative models for data sharing and international comparisons, for instance a federated approach for data collection, as shown in the current Research Topic “*Joining Efforts to Improve Data Quality and Harmonization Among European Population-Based Cancer Registries*” with the description of the Nordcan.R tool. The article showed how the tool is used to compute statistics for the Nordic cancer statistics web platform NORDCAN, and demonstrated that it works also with non-Nordic data (50).

An innovative approach in view of federated data quality evaluations was also presented in the current Research Topic. The article presented an ontology created using a modular approach to handle specific checks for childhood cancers, leading to a simpler maintenance of data validation rules (51).

In this context, a key role is going to be played by the future European Health Data Space (EHDS), a European Commission initiative to build a common EU framework facilitating the use of health data for secondary purposes that could be beneficial to European PBCRs by facilitating cancer data sharing (52). This initiative aims to improve interoperability and accessibility of health data across Europe, fostering better research and improved public health outcomes. By creating a standardised environment for health data exchange, the EHDS will enable more efficient data sharing between PBCRs and researchers, helping to overcome current barriers related to data fragmentation and diverse national regulations. This will not only help streamlining the process of data harmonisation but will also promote innovation in cancer research, ultimately contributing to more effective cancer prevention and treatment strategies across Europe.

Three articles in the present Research Topic focus on methodologies for the computation of cancer prevalence. A first article showed two alternative approaches in the framework of the completeness index method, based on incidence and survival modelling, in order to provide comparable indicators on complete cancer prevalence (53). The second article described the procedures to derive complete prevalence and several indicators of cancer cure from PBCRs. Limited duration prevalence was calculated for 62 cancer types by sex and PBCR, presenting indicators which may be relevant for patients and clinical practice and reproducible in different European countries (54). Lastly, a new method to estimate short-time projections of cancer prevalence by phase-of-care was illustrated. Evidence from this method was addressed to policy makers for planning future cancer care, thus improving cancer survivorship experience for patients and care-givers (55).

Finally, in recent years, biomarkers have become more important in guiding diagnosis and treatment options as well as for the prognosis of several tumour types such as, for example, breast, oropharyngeal and lung cancer (56). The use of biomarkers is also important in predicting recurrences. For this reason, biomarkers should be taken into account in the future by the ENCR because it will be necessary to standardise data collection, coding and reporting of this key information.

## Data availability statement

The original contributions presented in the study are included in the article/supplementary material. Further inquiries can be directed to the corresponding authors.

## Author contributions

FG: Conceptualization, Methodology, Project administration, Supervision, Writing – original draft, Writing – review & editing. CM: Conceptualization, Methodology, Supervision, Writing – original draft, Writing – review & editing. RC: Conceptualization, Project administration, Writing – review & editing. VZ: Writing – review & editing. OV: Writing – review & editing. MB: Conceptualization, Project administration, Supervision, Writing – review & editing. LVE: Conceptualization, Project administration, Supervision, Writing – review & editing.
